# The Lived Experiences of Men Who Engage in Online Child Sexual Exploitation and Abuse: An Interpretative Phenomenological Analysis

**DOI:** 10.1177/10790632261446953

**Published:** 2026-04-24

**Authors:** Grace Stewart (née Nock), Louise Dixon, Nichola Tyler

**Affiliations:** 1 School of Psychology, 8491 Victoria University of Wellington, Wellington, New Zealand; 2 3525 Pro Vice-Chancellor Education, Glasgow Caledonian University, Glasgow, Scotland; 3 Centre for Forensic Behavioural Science, 3783 Swinburne University of Technology, Hawthorn, VIC, Australia

**Keywords:** online child sexual exploitation and abuse, IPA, qualitative research, CSEM, online grooming, treatment, prevention

## Abstract

Online child sexual exploitation and abuse (OCSEA) is a complex and multifaceted form of offending that continues to increase in prevalence. Our understanding of OCSEA is challenged by evolving technologies, low rates of detection, and limited understanding of the aetiological pathways to engaging in this behaviour. The current study aimed to deepen our understanding of the aetiology of OCSEA through qualitatively exploring the lived experiences of nine men referred to New Zealand community treatment providers for OCSEA behaviours. Using Interpretative Phenomenological Analysis, men’s life stories were examined across the lifespan, including childhood, pre-offending, offending, and post-offending timeframes. Five superordinate themes were developed. These described the impact of the men’s developmental environment on their understanding of healthy relationships; the desire for connection and feelings of not belonging; difficulties in coping with negative emotions; escalation of both legal and illegal sexual behaviours; and factors associated with ceasing offending. The application of findings to the prevention of OCSEA are considered and directions for future research are discussed.

Online child sexual exploitation and abuse (OCSEA) is a global problem and reflects a complex suite of behaviours. Offence types are varied and include (but are not limited to) behaviours such as online grooming, child sexual exploitation material (CSEM; viewing, downloading, disseminating or creating), or engaging in pro-peadophillic forums. The dynamic nature of technology has had a corresponding impact on individuals exploiting new ways to offend, such as with AI facilitated child sexual abuse (e.g., artificially generated sexual images of minors that do not derive from real photographs; Internet Watch Foundation, 2024). While ascertaining the true extent of OCSEA is difficult, estimates suggest it is occurring on a large and increasing scale. Recent monitoring reports have documented substantial year-on-year increases in reports of online child sexual exploitation and abuse material globally (Childlight, 2024; Internet Watch Foundation, 2024), while meta-analytic findings across 123 studies identified a prevalence of OCSEA victimisation ranging from 8.1% for past-year recall to 16.6% for lifetime recall (Fry et al., 2025). Additionally, between 7–11% of men self-report engaging in OCSEA-related behaviours (Childlight, 2024). The staggering prevalence and continued growth of OCSEA must also be acknowledged in the context of the significant harms associated with this offence type, causing lasting trauma to victims (Hamilton-Giachritsis et al., 2020) and extensive costs to society (Giles et al., 2024).

Given both the incidence and harms associated with OCSEA, it is crucial that effective preventative strategies are employed using a multi-tiered public health approach (Baines, 2019). While interventions exist, including educational programmes (Patterson et al., 2022), warning messages (Prichard et al., 2022), and self-referred help for individuals concerned about their behaviour (Newman et al., 2024), there are limited evaluations that support the effectiveness of these programmes. Further, whilst growing, our understanding of OCSEA and the best way to prevent this remains largely understudied, with an overreliance on contact sexual offending research and convicted samples. If we are to advance effective prevention of OCSEA, we need to first invest in research that helps us understand the aetiology of the behaviour. Such qualitative accounts can inform prevention and treatment by identifying the specific beliefs, emotional processes, and interpersonal patterns that people themselves view as central to their offending, which can then be targeted in psycho-educational and therapeutic work. Without an understanding of the psychological mechanisms associated with OCSEA, our efforts to address this public health issue will remain largely based on guesswork and assumptions.

To date, research on OCSEA has tended to focus on quantitative examinations of the characteristics of individuals who engage in CSEM offences compared to contact sexual offending samples or mixed offending samples (Babchishin et al., 2015). In contrast, there has been little research that has qualitatively explored the experiences of people who engage in OCSEA. The lack of qualitative research in this area echoes similar knowledge gaps in forensic psychology more broadly, whereby only 11.3% of published research articles are of qualitative design (Copes et al., 2020). However, qualitative research is particularly useful for providing insights into people’s experiences, beliefs, and behaviours which can assist in the development of theory and generation of new hypotheses through an inductive understanding of the aetiology of the phenomenon being explored (Biggerstaff, 2012).

The few studies that have adopted qualitative methods when researching OCSEA have tended to focus on identifying behaviours, motivations, and cognitions associated with individuals’ offending from secondary data sources such as police interviews, forums, or chat logs (Black et al., 2015; Paquette & Cortoni, 2019). While beneficial, the use of secondary data introduces challenges with data quality, a lack of control over questioning, and limited contextual understanding (Tripathy, 2013). Research using primary data to directly examine the experiences of individuals who engage in OCSEA has predominantly emerged from UK samples. These studies often focus on specific psychological constructs such as the function of offending (Crookes et al., 2017), proximal factors associated with offending (e.g., offence-supportive cognitions, self-distancing; Kloess et al., 2019; Rimer & Holt, 2023; Winder & Gough, 2010), specific events (e.g., the impact of COVID-19 on sexual behaviours; McMahan et al., 2023), technical skills (Quayle et al., 2014), or the impact of specific treatment programmes (Dervley et al., 2017; Gillespie et al., 2018). However, there has been little focus on exploring the aetiology of OCSEA offending, or the interplay between various psychological factors, such as how cognitions develop, or the impact of offending on individuals’ non-offending lives (Soldino et al., 2019). Developing an understanding of the aetiology of OCSEA and how people who engage in this behaviour make sense of their experiences is important for advancing our understanding of how the offence process unfolds and identifying potential points for intervention.

There is only one study that has qualitatively examined the lived experiences of individuals who have engaged in OCSEA behaviour with a focus on both distal and proximal factors. In the current paper, we use *distal* to refer to developmental and contextual influences that emerge earlier in life (e.g., childhood abuse, attachment experiences, broader family and social environment), and *proximal* to refer to more immediate psychological and situational factors that are closer in time to the offending (e.g., mood states, sexual preoccupation, online opportunity). Using Interpretative Phenomenological Analysis (IPA), Sheehan and Sullivan (2010) examined the life stories of four men who had produced CSEM recruited through UK police forces. The authors identified a number of themes spanning early life experiences (e.g., childhood sexual behaviours, social isolation) and the impact of these on attitudes, sexual interests, and behaviours; obstacles and opportunities for generating CSEM (e.g., conflicted beliefs, offence supportive cognitions); and motivations for offending. Whilst this study enhanced understanding of some of the factors involved in the aetiology of CSEM manufacturing, participants were convicted individuals committing a specific and less commonly engaged in form of OCSEA. Given both the diversity and international reach of OCSEA offending, more qualitative research is needed to understand the motivations for offending online (Kloess et al., 2014), the distal factors contributing to OCSEA (Elliott & Beech, 2009), how different online offences interact or escalate (Kloess et al., 2014), and online grooming offences (Kloess et al., 2019). Quantitative studies have begun to identify a range of correlates associated with OCSEA, suggesting that men who offend online tend, on average, to be younger, relatively well educated, and to show higher rates of developmental adversity, intimacy deficits, loneliness, emotion-regulation difficulties, sexual preoccupation, and deviant sexual interests than non-offending or contact-offending comparison groups (Babchishin et al., 2011, 2015; Chopin et al., 2022; Henshaw et al., 2017; Middleton et al., 2006; Napier et al., 2024). However, this work has relied heavily on convicted or forensic samples, and has primarily focused on describing distal and proximal correlates rather than on how individuals themselves understand the aetiological pathways into their online offending.

## Current Study

The current study aimed to extend previous research by examining how individuals who have engaged in OCSEA understand and make sense of their offending with consideration to the role of developmental, psychological, behavioural, and contextual factors. Interpretative Phenomenological Analysis (IPA) was used to analyse in-depth individual interviews with nine men referred to New Zealand community treatment providers for OCSEA. By exploring the in-depth lived experiences of those who have engaged in OCSEA behaviours, it is hoped that the research will extend our understanding of the aetiology of OCSEA and highlight areas that can inform theory development and intervention.

## Method

### Participants

Nine men who had engaged in OCSEA were recruited through non-government treatment providers for harmful sexual behaviour in New Zealand. Individuals were eligible to participate in the research if they were male, over 18 years, and had a history of engaging in OCSEA including online grooming, sexual conduct with a minor facilitated through online technology, and using, downloading, or sharing CSEM. Participants were also eligible to participate if they had committed a contact offence alongside an online offence. The behaviour did not need to have resulted in a conviction for OCSEA, although it had to be known to authorities or the treatment agency. The sample size of nine participants is consistent with IPA’s idiographic and in-depth focus, which typically involves small, relatively homogeneous samples (often 3–10 participants; Pietkiewicz & Smith, 2014; Smith et al., 2021). This size allowed us to conduct detailed case-by-case analyses and then examine patterns of convergence and divergence across cases. Participants ranged in age from 24 to 56 years (M = 35.1). Most identified as heterosexual (*n* = 6), with the remainder describing themselves as bisexual, bi-curious, or gay. Eight of the nine men had at least one conviction related to OCSEA. The one individual without a conviction engaged in both online grooming and CSEM and were known to the treatment service and relevant authorities, in line with eligibility criteria. Six had engaged in online grooming, eight in CSEM use, and five in contact offending in addition to online behaviour (see Table 1).

**Table 1.** Participant Demographic Characteristics

Pseudonym	Age	Sexual orientation	Ethnicity	OCSEA conviction	CSEM	Online grooming	Contact offending	Previous conviction
Louis	39	Heterosexual	NZ European	Yes	Yes	Yes	Yes	No
Simon	24	Bi-curious	NZ European	Yes	Yes	No	No	No
Chris	26	Bisexual	NZ/South African	No	Yes	Yes	Yes	No
Jordan	39	Gay	Māori	Yes	Yes	No	No	No
Steve	31	Bisexual	NZ European	Yes	Yes	Yes	No	Yes
Liam	56	Heterosexual	NZ European	Yes	Yes	No	Yes	Yes
Tom	34	Heterosexual	NZ European	Yes	No	Yes	No	Yes
Phil	40	Heterosexual	NZ European	Yes	Yes	Yes	Yes	No
William	27	Heterosexual	NZ European	Yes	Yes	Yes	Yes	Yes

### Procedure

Ethical approval was obtained from the University of Wellington, Te Herenga Waka Human Ethics Committee (#30636). Research approval was also obtained from the New Zealand Department of Corrections, and two non-government organisations that provide intervention for harmful sexual behaviour in the community. Clinicians at participating services identified clients who met the study eligibility criteria and provided them with a one-page information sheet that briefly outlined the project. If potential participants were interested in learning more about the research, a full information sheet and consent form were provided. Informed consent was either obtained in writing or audio recorded prior to the interview. Participants were either able to contact the lead researcher directly to arrange an interview or to ask their clinician to pass along their contact details. Interviews were organised with consideration for both participant preference and licence conditions. Interviews took place either over the phone (*n* = 3), via videoconferencing (e.g., Zoom; *n* = 3), or in person (*n* = 3). All interviews were audio-recorded on an encrypted Dictaphone with participant consent. The interview duration ranged from 93 to 173 minutes (*m* = 116 minutes). Data were collected through a demographic questionnaire and a semi-structured interview schedule. The interview schedule was adapted from previous qualitative research exploring offence narratives (Tyler et al., 2014) and adopted a life course narrative approach to understand participants’ lived experiences of their offending behaviour. Interview questions explored participants’ childhood and developmental background, adolescence and early adulthood, the 6-month period preceding their first online offence until the last offence, interest and attraction to minors, and post offence experiences. Interviews were transcribed verbatim by the first author.

### Analysis

The study employed a qualitative design using a phenomenological approach (Smith et al., 2021). IPA was chosen as an appropriate methodology for analysing the data due to its ability to pay particular attention to participants’ lived experience of a phenomena, how they made sense of their experiences and the weight they attributed to these, centring their voice in the research (Eatough & Smith, 2017). Analysis was conducted according to the procedure outlined by Smith et al. (2021). Interviews were analysed individually for the first four stages, with group analysis occurring in step five and six:

#### Familiarisation

The lead researcher listened to each interview to familiarise themself with the data and refresh their memory on tone, utterances, and participant emotions. Interview transcripts were then read in conjunction with the audio recording and then re-read separately from the audio recording to note initial thoughts and observations of the data.

#### Exploratory Statements

Following familiarisation, transcripts were entered into a three-column table in MS Word. The first column contained the transcript, the second column was used for identifying and annotating exploratory statements and the third column was for experiential statements (next step). Exploratory statements sought to capture core ideas and experiences, participant idiosyncrasies, and the use of language, metaphors, tone, emotion, and conceptual questioning.

#### Experiential Statements

The third column in the word table was used to create experiential statements. Experiential statements were primarily based on a re-analysis of the exploratory notes, seeking to create a more concise summary of participants’ experiences. Experiential statements were then mapped in Miro (Miro is on online visual workspace used for creative collaboration and brainstorming), to find shared points of connection and meaning between the statements. The organisation of statements in Miro took several iterations and was a collaborative process between the lead researcher and supervisory team.

#### Personal Experiential Themes (PET)

The mapped-out experiential statements were then organised into PET, which provided a visual and descriptive framework for all the themes and sub-themes. To identify PET, the experiential statements were clustered in Miro to find areas of shared meaning, including superordinate and subordinate themes. These themes were then entered into a MS Word table wherein theme names were determined, alongside descriptions of each theme with supporting quotes. Each researcher reviewed the PET, and refinements were made to triangulate these with core participant experiences until consensus was reached.

#### Group Experiential Themes (GET)

The purpose of the GET is to identify the shared themes and sub-themes across participants. To do this, all the PETs were collectively analysed to find connections and meaning between each participant by looking for convergence or divergence in conceptual experiences. The group analysis was conducted collaboratively with the lead researcher and a supervisor through printing out each PET and manually comparing them across participants. To enable more flexible thinking, post-it notes were used to identify commonly occurring themes and ideas, which were then organised into posters representing each superordinate theme. This method allowed for a visual representation of the data and encouraged a more creative and flexible analytical process. Using these ideas, a GET was created, which was a table in MS Word stating the theme and subtheme name, description of each subtheme, and supporting quotes.

#### Write-Up

Writing up the results in IPA is an additional stage of the analytical process where further refinements were made as the full extrapolation of the results were identified. The process of writing the results allowed for themes to be fully developed and substantiated with supporting evidence and participant quotes. To capture individual differences, each case was considered independently, preserving unique perspectives within themes where experiences diverged. Divergent voices were woven into the narrative without forcing consensus, ensuring that each participant’s unique experience was authentically represented. Further, as theme descriptions were completely articulated, components of sub-themes moved to different sub-themes where the narrative and story of participant experiences flowed more clearly.

Given the interpretative nature of IPA, it is important to recognise the role of the researcher in this process and the potential impact of researcher bias (Biggerstaff & Thompson, 2008) and the need to be self-reflective (Eatough & Smith, 2017). The research team was comprised of three female researchers with academic backgrounds in forensic psychology, and whose previous research includes a focus on victimisation and perpetration of harmful sexual behaviours. The second and third author also have experience of working clinically in forensic settings including with men who have engaged in harmful sexual behaviours. Constant challenging of ideas was encouraged throughout analysis through regular supervision where different perspectives were considered and discussed. The third supervisor who had not been involved in creating the GET provided an independent review of the final GET for coherence. Themes were continuously cross-checked with the data at each stage of analysis to ensure they aligned with participants’ narratives, so their voices were centred throughout the process and that the meaning attributed to their experiences were reflected in the final themes.

## Results

**Figure 1.**
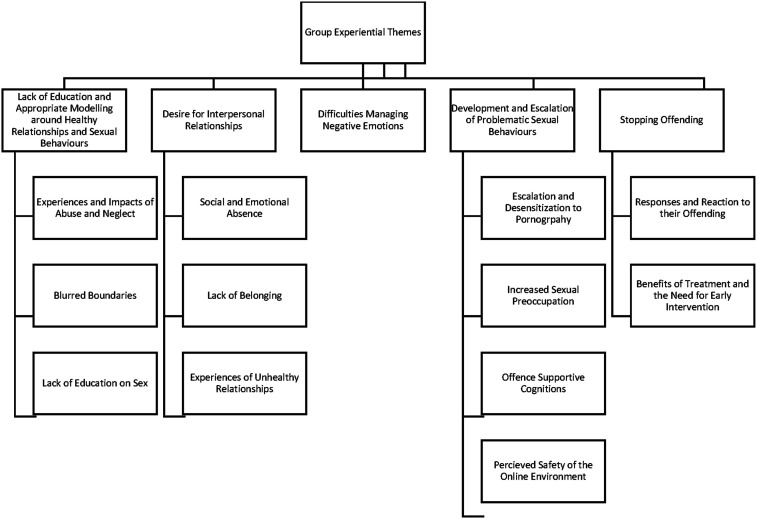
Representation of all superordinate themes and sub-themes

Five superordinate themes were developed from the data that described participants’ understanding of the thoughts, feelings, and experiences associated with their OCSEA offending: *Lack of Education and Appropriate Modelling around Healthy Relationships and Sexual Behaviours, Desire for Interpersonal Relationships, Difficulties Managing Negative Emotions, Development and Escalation of Problematic Sexual Behaviours*, and *Stopping Offending*. Figure 1 provides an overview of the themes and sub-themes. Each superordinate theme and their corresponding sub-themes are now discussed along with supporting quotes.

### Theme 1: Lack of Education and Appropriate Modelling Around Healthy Relationships and Sexual Behaviours

Participants discussed early formative experiences that contributed to both the development and reinforcement of problematic beliefs about relationships and sexual behaviour. Participants often described lacking the necessary guidance, education, or modelling to understand healthy relationships. Consequently, problematic interpersonal and sexual behaviours were normalised and provided participants with a skewed perception of safe relationships and sexual behaviours.

#### Early Experiences and Impacts of Abuse and Neglect

Most participants described experiencing some form of neglect and abuse during childhood and/or adolescence. Participants recounted experiences of sexual abuse, physical abuse, emotional abuse, and secondary trauma from witnessing abuse occurring between others. Over half of participants reported experiencing sexual abuse during childhood, which for some, extended into a pattern of sexual victimisation throughout adulthood. The experience of sexual abuse, and other people’s reaction to the abuse, impacted participants’ understanding and development of core beliefs about appropriate sexual behaviour, relationships, and consent that were reflected in participants offending later in life. The lack of appropriate intervention often normalised abusive experiences and led participants to feel like no one cared about them or their well-being. Participants reported receiving no support or guidance to process their experiences, reinforcing the idea that they were alone and what they were experiencing was acceptable. The normalisation of abuse led some men to believe that sexual abuse was an expected part of childhood and, as a result, viewed their own behaviour as acceptable.

Sexual abuse being the biggest one of it, and the way that my parents treated that sexual abuse, walking over it, acting like it didn't really happen, or acting like it was just something that happened built in the idea that it was something that happened to me, it was something that happened to other people… that people that just experience. So when I access it online, I was just like, oh, someone was just filming this experience... It wasn't something that outrightly I looked at was wrong. – Chris

The experience of sexual abuse at such a formative stage was reported to contribute to an early awareness of a sexual attraction towards children for some of the men, as Chris described, “As a result, like afterwards, it’s created a bunch of negative sexual interests, which is uh problematic, to say the least.”. For others, such as Jordan, these experiences impacted the development of healthy relationships, with some participants developing a belief that sexual activity was the only way to connect with others, “I tried to get connections with people, but it just, always with gay guys, but it just always ended up being in the bedroom rather than in person.”

Witnessing aggression in the familial context also impacted the development of unhealthy attitudes towards conflict in relationships and sometimes led to mirroring of aggression in participants’ adult relationships or the use of aggression to connect with others. This behaviour was particularly relevant for Simon, who recounted “As early back as kindergarten I was, you know, pushing kids down the slide, because it was the only way that people would actually interact with me…”

#### Blurred Boundaries

Most participants described lacking boundaries growing up and an absence of modelling and reinforcement of appropriate social and sexual behaviours. Parents were often described as providing poor supervision or oversight of behaviour, resulting in participants engaging in antisocial behaviour, truanting, or leaving home early. Jordan experienced this parental disconnect acutely, “Like, I was 17, living in an apartment with drug addicts.”

Parents were also reported to have provided insufficient guidance on normative social behaviours and values, or how to interact with others safely. Participants attributed their difficulties with forming interpersonal relationships to the lack of parental modelling and boundary setting. Participants often felt they missed social cues or were seen as “weird” by others due to their use of problematic behaviours, such as asking personal question to form connections.

I was never taught what was right and wrong, and I always took things too far with my family…I was never taught boundaries. What is ok and what is not ok… it's different for each family, but for me there was like no boundary for me and I wonder if I was younger I might have been taught more about boundaries and social cues, more. - William

Participants discussed how sexualised behaviour was often normalised in the familial context from a young age. For example, some participants reported engaging in problematic sexual behaviours during childhood which went unchallenged or without explanation as to why it was inappropriate, “Um but I still never really saw of it as something that was wrong. It was never taught to me that right, wrong. It was just no, don’t do this.” Other participants discussed being exposed to adult concepts at a young age, for example, through exposure to sex toys, pornography and nudity at home. Participants perceived these experiences as contributing towards both the normalisation of sexual behaviour as well as them perpetuating harmful behaviours towards other children.

While most participants described an upbringing characterised by a lack of boundaries, Louis reported his parents were very strict and would not allow him to play or explore. This restrictive upbringing meant Louis felt he lacked a safe environment to learn and make mistakes, which impacted his sense of self and experience of being trusted. The feeling of being untrustworthy was perceived to impact his ability to build authentic relationships and connect to others, “I grew up feeling that dad didn’t have the confidence and he didn’t trust me. So then that thinking, became my thinking. I’m not good at this and I’m not trustworthy.”

#### Lack of Sex Education

Almost all participants described a lack of sex education growing up, which they believed exacerbated problematic attitudes surrounding sex and relationships. Participants often described their parents as being uncomfortable teaching their children about puberty, sexual development, abusive experiences, and healthy sexual relationships. Without an open dialogue or education around healthy sexual behaviours, participants reported they did not always understand that some of their early sexual behaviours were inappropriate or illegal or that their foundational beliefs surrounding sex and relationships were harmful.

I mean, most parents would want to talk to their kids about what happens during puberty. My parents, I think it again, they're of the older generation. So, you know, it wasn't really a done thing. So, all of a sudden, I'm in a sex-ed class and I'm like what on what on earth is this all about? – Louis

Appropriate sex education was also reported to be lacking in the school curriculum by many of the men. The paucity of sex education was perceived to limit opportunities for modelling of healthy sexual behaviours and to challenge problematic attitudes and beliefs about sex and relationships that developed through participants’ early exposure to sexual content and abuse. The lack of school-based sex education was experienced by many of the men as impacting their understanding of consent and healthy sexual relationships, “…I never learned about sex-ed at all. We didn’t learn about sex at primary school, I didn’t learn about it at high school” (William), “When I was in school, consent wasn’t something that was really discussed” (Chris).

A lack of understanding of consent contributed to offending behaviour by the men as they did not understand boundaries within sexual relationships. Early problematic behaviour towards others, or inflicted on them by others, was considered normal. Likewise, other problematic influences such as pornography were seen as a source of guidance for healthy sexual interactions.

### Theme 2: Desire for Interpersonal Relationships

Participants reported a strong desire for social, familial, and romantic connections, however, frequently experienced challenges in obtaining these. Difficulties in adolescent and adult relationships were often attributed to early experiences of disconnection at home and problematic beliefs surrounding healthy relationships and sex, which supported offending behaviour. Participants desired connections but often felt excluded, emotionally disconnected, or different from peers, family, and potential romantic partners. When relationships were created, these were often characterised by physical and emotional abuse which enhanced the experience of isolation and feeling of marginalisation. Whereas Theme 1 focused on the developmental context and modelling that shaped participants’ early beliefs about relationships and sexuality, Theme 2 centres on how they later experienced connection, belonging, and intimacy across adolescence and adulthood.

#### Social and Emotional Absence

All participants encountered some degree of social and/or emotional disconnection from their parents. Parents were frequently described as rejecting or diminishing the emotional well-being of their children, which was believed to impact upon participants’ ability to understand and express their feelings or learn healthy help-seeking behaviours. Participants often reported developing the belief that their needs were not important. The lack of consideration given to participants’ emotional and mental well-being growing up was seen to impact their sense of self-worth, making them feel uncared for and unworthy. Chris described this impact on his self-worth, “so, it gave me an impression that my feelings don’t matter, what I think and feel doesn’t matter. So why should I bother talking about it if it doesn’t matter?”

The disconnection from parents and caregivers was described by participants to also lead to feelings of abandonment and betrayal, such as Phil’s experience of being “Very abandoned, extremely abandoned.” The lack of reliable and safe early attachment to parents was subsequently viewed as negatively affecting the men’s ability to trust others and created recurring fears of abandonment.

#### Lack of Belonging

Participants often described feeling like they did not fit in at home or with peers. Feeling different or abnormal corresponded with a desire to fit in and be accepted by others and was associated with attempts to find a sense of belonging and connection to others. “As a young kid, it felt like, as ridiculous as it sounds now, it felt like there was this pact that everyone had signed to intentionally ignore the weird kid” (Simon).

Lack of belonging manifested in numerous ways, including a lack of friendships, feeling different or excluded, or being bullied and rejected. Steve was repeatedly bullied growing up, which heightened his feeling of isolation and difference from others, “Yeah, just almost constant bullying and didn’t really have any friends.”

For some participants, such as Liam, moving frequently within New Zealand or internationally during adolescence was associated with difficulties in forming and maintaining friendships and feelings of loneliness, “yeah, but you know, it was hard because I’d make friends and then we’d move and then I’d have to make new friends.”

For Jordan, the lack of belonging extended beyond social and familial spheres to include cultural disconnection. He expressed a sense of being whakawehe^1^, being alienated or set apart from his whakapapa^2^ and whānau^3^. Jordan’s disconnection from his Māori heritage along with the complexities of navigating his sexuality during adolescence, whilst also experiencing a lack of social or familial belonging exacerbated his struggle with his identity. “They’re all Māori, into their Māori tanga, I’m just, I’m not.”

Due to the lack of belonging within their social and familial environment, participants reported experiencing a strong desire to develop connections with those around them. For Chris, finding a friend was highly desirable, as he wanted to open-up to someone fully and build an enduring bond, “I think that’s a similar factor to almost everything in my life, finding someone to connect to.”

William primarily desired a romantic connection; however, this desire was so overwhelming that he would frequently engage in problematic behaviours to obtain this, including sending unsolicited nude photographs, stalking, lying to women, or trapping women into being in a relationship with him. “I had a connection with her and I, everyone I’ve noticed, everyone I have connection with, I take it way too far or I think the connection is a lot stronger than the normal.”

Difficulties in forming connections and communicating effectively with people was commonly reported by participants. A lack of emotional intelligence and understanding of social dynamics often led to individuals building superficial friendships with problematic influences. These influences included engaging in antisocial behaviour such as substance use, skipping school, or getting into trouble with the police. For example, Tom described, “They were the wrong couple of friends I should have met. But at that time, you know, I didn’t have anybody, so anybody that… I was, I was into cars and they were into cars so, you know.” The negative influence of superficial relationships was further compounded by the disconnection experienced from parents, for example, through being kicked out of home.

A desire for connection and difficulties in obtaining this, meant that participants often turned to the internet as a way to connect with others, viewing this as a safer environment within which to achieve belonging. Participants reported spending increasing amounts of time online to experience a place of acceptance. Some of the social difficulties experienced by some participants like William were attributed to suspected or diagnosed neurodiversity, such as autism, “The autism diagnosis but then getting help for that and working out boundaries and working out how to be appropriate online. I’ve never learned how to be appropriate online ever.”

The absence of skills needed for building relationships with similar age peers also appeared to lead to a belief that children were safer than adults for some of the men. After experiencing repeated disconnection, rejection, and difficulty communicating with people of similar ages throughout life, some men such as Louis then turned to children for relationships, “I would have said that 12 to 16 was easier, easier online. I think, talking to adults online, it’s possible that because of, I tried it, it’s possible that they saw me as immature. So it never worked. And I saw them as, the Trunchbull.”

#### Experiences of Unhealthy Relationships

Experiencing and witnessing problematic relationships, coupled with a strong desire for belonging and connection was associated with almost all participants engaging in unhealthy romantic relationships during adolescence and adulthood. Toxic and mutually abusive relationships were common and contributed to feelings of isolation, emotional separation, and being unsafe. In response to these experiences, participants reported seeking out other avenues to feel in control and safe, for example, through offending against minors.

I was quite worried I had attraction to young kids. I was really quite worried about it. But I don't, I have an attraction to people that give me what I want. Bit of like a power thing. I think I've got a bit of a control issue. - William

Participants would try to protect or buffer themselves from the negative feelings that accompanied failed relationships through engaging in pre-emptive sabotage, cheating, keeping emotional distance, or focusing purely on sexual connection. However, these avoidant behaviours often prolonged their feelings of loneliness as they were not able to build healthy and trusting adult relationships. “So I kinda get, I kinda get the sexual need met but I just didn’t have the tools to have a close relationship for any kind of long term period” (Liam).

Despite the fears and expectations of negative relationships, participants deeply desired romantic connections and wanted them to succeed, “…and I was praying, I was hoping like hope it turned, it doesn’t turn to shit, you know, like all my other relationship” (Tom). The need for romantic acceptance meant the men would often ignore “red flags” in the women they dated or would pursue people with obsession to try and obtain romantic connection.

### Theme 3: Difficulties Managing Negative Emotions

Participants commonly reported experiencing difficulties with negative emotions and lacked effective coping strategies to manage these. Around half of participants reported a diagnosis of a mood disorder (e.g., anxiety, depression or bipolar) or a history of suicidal behaviour. While the remaining participants did not have a formal diagnosis of an affective disorder, they all reported feelings of depression, sadness, anxiety, loneliness, stress, and rejection.

I was just really lonely. I just wanted to talk to people. I'm like, I'm just gonna talk to people no matter what. I was down. I felt sorry for myself. I'd have suicidal thoughts. I wouldn't sleep. - William

Participants lacked effective coping strategies to manage negative emotions, having learned from their previous experiences with peers, family, and romantic partners that they could not seek help from others or rely on them for support. Instead, including Steve and Liam, described employing avoidance or emotion-focused coping strategies to manage emotional discomfort, including substance use, “the drugs made me happy, and the alcohol helped me to forget,” mental distancing, running away, self-harming, violence, or withdrawing from those around them, “I just, you know, started living in a fantasy realm to protect myself from the abuse.”

Sexual behaviours, including, legal pornography, accessing CSEM, and communicating with minors online, were also reported as strategies employed to cope with negative affect. Feelings of boredom, loneliness, frustration, or depression often preceded individuals’ offending and the online world was perceived as a place to escape these feelings.

Yeah, usually at periods where I felt extremely lonely and extremely bored, yeah. That's where, though, especially the loneliness that's when it would come on, you know, when I'd be sitting there, it would be like 10 at night I had nothing, nobody to talk to, nothing to do, you know, and I just felt like I just wanted some sort of hit, you know, some sort of exhilaration. – Phil

The use of poor coping strategies however, often exacerbated negative mood states for the men, impacting upon the development of spiralling and negative emotions and behaviour. While offending provided temporary gratification, the immediate post-offence experiences tended to exacerbate negative emotional states, wherein individuals felt guilty, disgusted, or instances of self-loathing by their actions online, “I definitely hated myself afterwards.”

### Theme 4: Development and Escalation of Problematic Sexual Behaviours

This theme describes participants’ sexual development, and the impact of early exposure to pornography on sexual preoccupation and dependency. This early preoccupation then impacted upon sensation seeking, sexual fixation and problematic attitudes, that escalated over time and reinforced offending behaviour. The changing nature and role of pornography, the role of the internet, an increased focus and interest on sex, and the presence of attitudes that support legal and illegal sexual behaviour appeared to influence both the continuity and escalation of problematic sexual behaviours. In this theme, *escalation and desensitisation to pornography* refers to increases in frequency and extremity of material over time, *sexual preoccupation* to the degree to which sexual thoughts and behaviours dominated participants’ time and attention, *offence-supportive cognitions* to the beliefs and justifications that permitted or minimised engagement in OCSEA, and *perceived safety of the online environment* to participants’ sense of anonymity, disinhibition, and reduced likelihood of consequences.

#### Escalation and De-Sensitization to Pornography

A pattern of escalation in both the frequency and content of online pornography and a corresponding desensitisation to material was commonly reported by participants. Exposure to adult pornography at a very young age was a shared experience and participants rapidly developed a level of dependency or a sense of addiction to legal pornography. The routine of seeking and consuming pornography often became habitual for participants and was reported to shape attitudes surrounding women and the nature of sexual relationships.

And I think that [pornography] irrevocably changed my whole perception of sexual relationships and females and stuff from a very young age. I had a completely irrational misconception of what sex actually was and what relationships were, and it went from watching them [pornography], you know, once a week to pretty much craving to watch them as soon as I finish school. I'd be excited about getting home to watch stuff. - Phil

As participants consumed increasing amounts of legal pornography, they began to become desensitised to the content, not experiencing the same level of excitement or arousal from it. There was often then an escalation in frequency and content sought by men, as participants then began to seek out new content or sexual interactions (e.g., paying adults for content) which tended to be more extreme in nature.

Growing up in teenage years as a teenager, dial up Internet, mum's not home, in the afternoon, you'll find stuff to look at. And then it's not enough. And you find people to talk to, and it's not enough. – Louis

The process of seeking out new or more extreme content, appeared to provide a gateway to accessing and using CSEM material for many participants, although, it is important to note that two participants did not search for CSEM. While accessing CSEM was part of an escalation of more frequent and extreme pornography use for most participants, (e.g., exposure to pornography containing bestiality), some accidentally stumbled across CSEM while seeking legal content, “I don’t think it was until I was 19/20 that I discovered the underage side of things. And it was more that I kind of just stumbled upon it” (Chris), and one other participant first accessed CSEM while trying to find videos of his own sexual abuse.

The first encounter with CSEM sparked a range of reactions from participants, including curiosity, an awareness of harm, immediate sexual desire, or a feeling of power in the knowledge of offending.

The first time I ever viewed it, I was expecting the cops to sort of kick down the door right at that second. And then when it didn't happen, you sort of feel a little bit invincible for a time. - Simon

As CSEM use increased, most participants reported a continued need for content to be more intense or severe in nature, either through the age range or depicted acts. As Simon describes, “yeah, the age range got wider. It was, you know, but only sort of wider backwards. So stayed at maybe 16 tops and then sort of went all the way back to like five and six.” This process reflected a habituation to material with more radical content required to meet the same adrenaline needs.

Two participants did not actively search for CSEM. Tom’s offending involved online grooming and Phil engaged in contact offending generating CSEM through the sharing of images as part of this. While the modus operandi of their offending was different to other participants, Tom and Phil experienced similar escalatory and desensitising behaviour in relation to their pornography use as those who accidentally or intentionally accessed CSEM, with Phil commenting on the difficulty in preventing escalation of online sexual behaviours to CSEM use.

Oh my god. I just, I couldn't have brought myself to that level. But I can absolutely understand, and I'll tell you what, it took a hell of a lot of control. It took a hell of a lot of control not to go to that level, but with the stuff that you can view. And the stuff that's on there, it drives you to that point, it instils in your mind that it's okay. - Phil

#### Increased Sexual Preoccupation

Alongside the escalation in the frequency and severity of their pornography use, many participants also reported experiencing a marked increase in sexual preoccupation. Sexual interests became a dominant focus occupying much of the men’s time with them actively seeking out opportunities to engage in sexual behaviours. For example, participants would spend long periods of time online and/or masturbating, and seeking out sexual content online would supersede relationships, work, and health.

I had no job and I used to spend all day online and by all day. I mean like, I used to masturbate four or five times a day. I'd send pictures all day. I'd be like the biggest bum. I'd ordered maccas all day. I wouldn't get out my room and ate, only time I’d leave my rooms was to go to the toilet. - William

Other participants reported seeking out opportunities to be alone so they could access pornography and CSEM. The desire for new content was so great that individuals would lie and borrow money from family to access material. The insatiable need for sexual gratification was likened by Phil to a basic survival instinct, “… it would be something that I’d just I guess look at, satisfied, and then done. Now I can move on with life. It’s almost like I was, I need to be fed.”

The impact of this sexual preoccupation coupled with the problematic attitudes towards sex and a desire for connection, outlined in the earlier themes, led to some of the men focusing on sex to build intimacy and or connection with others “because I had been having unprotected sex since I was 18/19 with, like, let’s say maybe three or four people a week.” The focus of sex to form relationships occurred primarily either online through sexual chatting, grooming and/or pornography use, or offline sexual connection with others.

#### Offence Supportive Cognitions

Participants expressed a range of offence supportive attitudes and permission-giving thoughts which facilitated and/or justified their engagement in OCSEA. While some of these attitudes emerged from early childhood experiences (e.g., abuse and normalisation of behaviour through caregivers’ reactions), other attitudes developed through pornography use. Many participants had developed the belief that children were able to consent and enjoy sexual activity. For example, Louis commented on how “happy” children looked in CSEM, whereas William perceived one young person he was exchanging images with had the capacity to know what they were doing, “this is her doing and I sort of blamed her because I sort of felt like she knew what she was doing. Because she was quite intelligent.”

Participants also minimised the harm of offending. The availability of CSEM was considered by many of the men to represent the legality of material as Louis stated, “…if this is illegal, why are you… why can I access it so easily?” Likewise the online environment and viewing of material was often seen as not as real or harmful, “I kind of not, had started viewing anything that was online as being as not being real, I didn’t really have a thought around not viewing it.”

Although participants reported a range of offence supportive beliefs, many also recognised that their behaviours were wrong. Some participants reported feeling conflicted between their need for sexual gratification and their moral reactions regarding the nature of the material they were accessing, such as for Tom, “I knew it was wrong. But my lust for it was higher than that, you know…” This conflict appeared to be resolved through a process of moral disengagement, whereby participants separated their moral reactions from their behaviours, allowing their sexual desires to prevail.

Despite engaging in behaviours they knew were wrong, some men perceived those who produced or shared material as “disgusting,” distancing themselves from these individuals even though they were also engaging in the behaviour themselves, drawing a “them” and “me” distinction. For example, Simon perceived his actions as different and more benevolent than those of other people who were offending, “I was quite disgusted by the people that were sharing those links, despite being one myself.”

Although Phil’s offending pathway looked slightly different to that of other participants (i.e., engaging in contact offending which transitioned into the sharing of messages and images online with the same victim), he shared similar offence supportive attitudes to other participants; for example, minimising the harm caused by his actions as not as “full-on” as other forms of contact abuse. Online offending was also seen as less harmful, described as a preparatory step to offending rather than being an offence in and of itself.

Yeah, to me there's a huge, huge difference between the two. You know, there's there's a thought of doing something compared to actually doing something, you know somebody can, somebody can buy a gun and take a gun with them, but it's not till you shoot someone that the gun becomes a problem. – Phil

All of these attitudes appeared to support and perpetuate the offending process for participants.

#### Perceived Safety of the Online Environment

Due to feeling socially excluded and different from peers and family, participants often perceived the offline world as unsafe for connecting with others or being their authentic self. Comparatively the online environment provided a sense of anonymity and provided the men with the opportunity to feel accepted by being the person they wanted to be and/or have the relationships they wanted to have. Simon did not feel he could build connections based on who he was, and rather, had to adopt a different personality to connect with someone, “the internet as a whole was and still is a place where I could be someone else. You know you could have 27 different personalities on the internet and no one bats an eye.”

it's very easy to hide behind a phone … if I put myself out there a lot of people find me weird. So like face to face, I'll say something they're like ‘that's weird”, I'm gonna walk away. Where online you can pretend to be something else … – William

The perceived safety of the online world also influenced participants’ offending, creating an environment where they felt safe to behave in ways they would not offline due to anonymity the internet provided, and a perceived lack of repercussions. The lack of immediate punishment for their behaviour created feelings of safety and, for some, feelings of invincibility, “I definitely thought I was, I could get away with anything” (Simon).

However, for some participants like Chris, using CSEM or speaking to people online was also considered a risky activity, as it may increase the likelihood of further offending, including contact offending, “a terrifying, terrifying thought and when I thought of, this is something that could lead to something in person, was a terrifying thought.”

While Phil did not access CSEM, he also viewed the online environment as potentially enabling more harmful behaviour, “I strongly believe that anybody who views the underage stuff has the potential to then go further. Absolutely, without a doubt. And the only reason they probably don’t is because they don’t have the opportunity.”

### Theme 5: Stopping Offending

This theme describes participants’ reflections on stopping their offending and the impact that their offending behaviour had on their relationships, the role of treatment in supporting the development of insight into their behaviour, and the need for early intervention to reduce the incidence of OCSEA. While the preceding themes focus on factors contributing to the development and maintenance of OCSEA, Theme 5 considers how participants made sense of stopping their offending and engaging in treatment. These reflections illuminate how they understood and responded to earlier vulnerabilities, and highlight potential points for intervention along the offence pathway.

#### Responses and Reactions to Their Offending

Participants experienced mixed reactions to being caught and stopping their offending. For some, this led to them feeling “completely panicked” (Tom) by the realisation that their family and the wider community would become aware of their behaviour. The fear and panic experienced was related primarily to how others would perceive them in light of their behaviour online, and the impact it would have on their relationships.

The amount of stigma around paedophilia. I know how wrong it is and I know that if she's calling me it, it's possible that it could be true and that if anybody else found out anybody significant in my life, like if my parents or if work or anything else found out then…. It could completely ruin my life. - Chris

In contrast, Phil experienced a sense of emptiness knowing the validation he experienced through offending would now cease, “but I also felt a bit of emptiness, as well, you know, like I’m returning back to, what it was like.”

More commonly, the men reported experiencing relief at being caught as they could now stop engaging in sexual behaviour they previously felt they had no control over, or was a “burden” to them. Liam acutely felt this relief at getting caught, “I knew I was gonna get caught eventually but I couldn’t stop it and so I felt relief, really. Relief that it was out in the open.”

Beyond their own experiences of fear, emptiness, or relief, participants’ offending often had negative impacts on interpersonal relationships including loss of peers, family, and breakdown of adult intimate relationships. Disclosing their offending to family and partners was experienced as particularly challenging as it often precipitated the breakdown of these relationships, acting as either a “wake up call” for participants like Tom “my wife was pretty upset. There was talk of divorce and stuff, if I’d gone to jail she would have left me, so it was a bit of a wake-up call.” Conversely, other men such as Steve were left feeling isolated, “I rang my mum probably about a couple of hours after the police left with the devices and she hung up on me…. Yeah, that was sort of expected but still like a slap in the face.”

However, when family and friends stood by participants’, this acted as a protective factor reducing feelings of isolation, supporting well-being, and creating a safe environment for participants while navigating treatment and rebuilding their lives. “My mother’s been very, very good. My sister’s been really good. And I even had a girlfriend. We were together for a year who supported me for the entire process” (Phil).

#### Benefits of Treatment and the Need for Early Intervention

The men reflected on how treatment had helped develop insight into their behaviour and supported them to develop skills in emotion regulation, taking accountability for their actions, managing mental health, and building healthy relationships. Louis had historically placed blame on others for his actions, which was a key learning process in his healing journey “for the longest time I’ve blamed them [parents] for who I am. And then it was only three or four weeks ago, that the penny dropped. Yeah, well, I’m my own person…”

Treatment was also seen to provide an opportunity to process unresolved childhood trauma, supporting the men to understand the impacts of developmental experiences on their relationships and beliefs about intimacy.

I had some baggage I needed to deal with from my childhood … I still kind of am a bit self-destructible, but I've been doing counselling for that like just balanced thinking and cognitive behavioural therapy, and the stuff at Stop around intimacy and that sort of thing. - Liam

Through treatment, participants reported developing a better understanding of consent, boundaries, healthy sexual behaviour, and how online offending is not a victimless crime, challenging previous offence supportive cognitions.

Since going to therapy, it changed my mind view on it, that I realised that there were other people on the other side of this camera that they weren't just objects, that they had lives. They were the ones who were being affected. That the way that I'm affected due to sexual abuse, they are also affected by sexual abuse. - Chris

Treatment also provided an opportunity to reflect on the need for early intervention. The need for early intervention to reduce people engaging in OCSEA was strongly supported by many participants. Consistent and comprehensive sex education in schools was considered important to provide guidance on consent, boundaries, and safe online behaviour. Participants also highlighted the need for early intervention with young people displaying problematic sexual behaviours to provide guidance on how to build healthy and safe relationships, and to redirect individuals from a pathway of engaging in repeated problematic behaviours. William was able to reflect on this need for early intervention, especially in light of his own problematic behaviours emerging at such a young age, “but I believe that people need, like me in particular, need help earlier. Like I wish I could go back and change my life, but I can’t.”

Community-based support services were viewed positively, with early intervention considered crucial for addressing sexual desires or concerns about escalating behaviour. Yet, many were unaware of services in the community that people could access. Even if participants were aware of services, stigma and potential legal repercussions were cited as a barrier to help-seeking, inducing fear of embarrassment or possible law enforcement intervention.

There was periods of my life where I did try to stop and I just couldn't. I tried referring myself to mental health services, but I was a bit scared about being completely honest with why. Or what it was that I was viewing so, I tried to send myself to Safe at that point, but because I was very vague about why, they referred me back to mental health services. - Steve

Participants also emphasised the need for increased awareness regarding the harms of pornography on sexual interests, attitudes to women and others, and habituation. The use of warning messages and stricter censorship of problematic content (e.g., ‘barely legal’ or ‘teen’) were also suggested as ways to reduce exposure to harmful content that can escalate and increase the awareness of the harms of such content. “Sites like Pornhub… when you’re going into these categories there should be some sort of warning that comes up about the impacts of what you’re watching,” (Phil).

## Discussion

This study investigated the lived experiences of nine men who had engaged in OCSEA. Using IPA, we examined how participants’ made sense of their offending through exploring in-depth the role of developmental, psychological, behavioural and environmental influences throughout life. Five superordinate themes described the impact of the developmental context on attachment, attitude and relationship formation; the desire for and difficulties forming romantic and platonic relationships; problems coping with difficult emotions; escalation and desensitisation to legal and illegal pornography; and the process of stopping offending. Together, these themes map a developmental and psychological pathway in which early adversity and limited corrective modelling shaped beliefs about relationships and sex, later difficulties with belonging and managing negative affect increased vulnerability, and escalating sexual preoccupation and offence-supportive cognitions were channelled through a seemingly safe online space, culminating in OCSEA.

The developmental context was perceived by participants as pivotal in shaping their attitudes and behaviour towards sex, relationships, and offending. The experiences of abuse, particularly sexual abuse, and the corresponding lack of social and emotional support by parents or caregivers was perceived to inform the formation of beliefs surrounding relationships, the self-concept, and attitudes regarding sex. Previous research suggests that individuals who commit OCSEA experience elevated rates of childhood abuse relative to non-offending samples (Babchishin et al., 2011; Napier et al., 2024), with the experience of child-to-child sexual behaviour also prevalent (Howitt & Sheldon, 2007). However, consideration of broader developmental experiences and the environmental context in which the abuse occurs has received less empirical attention. While research has identified the impact of victimisation on subsequent criminogenic needs (Chopin et al., 2022), including the role of normalising abuse or offence supportive attitudes (Grady et al., 2022), the impact of social support in response to abuse should be examined further. A lack of support from parents in the aftermath of abusive experiences has been found to significantly relate to poor adjustment, psychological distress, and anxious attachment (Godbout et al., 2014). In contrast, when effective support from parents is provided, it is influential enough to even lead to improved attachment outcomes for children, specifically reduced instances of avoidant attachment, as compared to non-abused individuals (Godbout et al., 2014). The role of parents in mediating the aftermath of abusive experiences is complex, and in our sample, the impacts of childhood abuse appeared to be heightened by poor caregiver responses, poor boundary setting, and a lack of alternative messages to challenge the development of problematic attitudes. The current findings indicate that a socio-ecological approach to prevention that considers the impacts of the wider familial and developmental environment is required.

Social and emotional rejection by peers and family, and a subsequent desire for connection and belonging was associated with participants seeking out safety and belonging in the online environment. Social belonging and connection have received less attention in the literature than romantic connection. However, some qualitative research has identified a desire for platonic connection as a motivation for engaging in CSEM use (Merdian et al., 2021; Sheehan & Sullivan, 2010). While the desire for social connection does not appear to be a direct motivation for offending, it may indirectly lead individuals to seek out like-minded individuals online (Elliott & Beech, 2009; Steel et al., 2023). In the current study, social isolation appeared to increase time spent online interacting with others as well as the opportunity to offend without detection.

Several participants discussed how their difficulties forming connections with peers reflected wider communication and interpersonal challenges. Social skills training is commonly recommended in treatment for OCSEA, reflecting known challenges for this population (Armstrong & Mellor, 2016). Several participants attributed their communication challenges to self-identified or diagnosed neurodiversity, such as autism. While there is not a causal relationship between neurodiversity and OCSEA offending (Allely & Dubin, 2018), it is important to understand how expressions of neurodiversity may present as vulnerabilities for some people’s involvement in OCSEA. For example, Allely and Dubin (2018) suggest that a lack of awareness of the illegality, the nature of collecting behaviour, and social immaturity leading to connections with younger people are often implicated in the offending pathways of autistic individuals engaged in OCSEA. Likewise, social skills difficulties, a lack of relationships, or not appreciating the harms of their behaviour, are suggested motivators for autistic people who sexually offend (Payne et al., 2020). In our study, the association mostly appeared to correspond to the perception children were relatable rather than the nature of harm or collecting, however, this belief was also shared by individuals who did not reference neurodiversity. Similarly, poor social skills and minimising harms associated with offending also occurred across the sample, suggesting that these domains likely represent shared areas of vulnerability for people who engage in problematic online behaviours regardless of neurodiversity. For example, experience of loneliness has been found to predict deliberate engagement in CSEM users more broadly (Napier et al., 2024). Therefore, prevention and intervention initiatives that target these domains of psychological vulnerability more broadly are likely to be most beneficial.

Difficulties in forming healthy romantic relationships in adulthood were commonly cited by participants in the current study. Intimacy deficits have frequently been identified among individuals who commit OCSEA and sexual offences more generally (Merdian et al., 2018; Middleton et al., 2006) with individuals often having few partners (Laulik et al., 2007) or lacking a partner at the time of offending (Babchishin et al., 2015). Participants in the current study did not necessarily lack relationships, but experienced difficulties in building or maintaining healthy romantic relationships, which acted as a catalyst for their offending. These findings align with existing theories of OCSEA (e.g. Pathways Model; Middleton et al., 2006) which suggest that intimacy deficits may contribute to offending behaviours by leading individuals to seek alternative, often inappropriate, avenues to fulfil unmet relational or emotional needs.

Difficulties in coping with and responding to negative emotions, particularly in relation to loneliness and relationship problems, appeared to be a common precursor to offending, and has been found to be predictive of online offending (Napier et al., 2024). Extant research suggests those who engage in both online and contact sexual offending experience difficulties with managing negative emotions (Paquette & Cortoni, 2021; Quayle & Taylor, 2003). The current sample used a myriad of maladaptive coping mechanisms to manage emotions. However, specific strategies employed are rarely explored in detail (Dervley et al., 2017). One clear maladaptive coping mechanism identified in both the current study and previous research, is the role of sex as a coping strategy (Babchishin et al., 2015). Emotion- and avoidance-focused coping broadly were particularly prevalent in our research, aligned with findings on contact sexual offending (Marshall et al., 2000). Additionally, the inability to effectively manage negative emotions appeared to be a proximal trigger to offending for participants in the current study, wherein individuals perceived the internet as providing a way to manage low mood. However, the increased time online further damages affective functioning (Laulik et al., 2007). This study, along with previous research (Bergner-Koether et al., 2024), suggests a negative behavioural cycle wherein participants use the internet, CSEM, or online grooming to cope with mood states, only to feel worse post-offence, reinforcing a circular pattern of behaviour. Interventions should therefore equip individuals engaged in OCSEA with effective self- and emotion regulation strategies, such as task-focused coping compared to emotion-focused coping.

Sexual preoccupation and an escalation and subsequent desensitisation to pornography was common in participants’ pathways to offending. Previous research has found CSEM users have high levels of sexual preoccupation or hypersexuality, associated with excessive time spent pursuing sexualised goals (Babchishin et al., 2015; Paquette & Cortoni, 2021) as compared to individuals who engage in online grooming (Seto et al., 2012). Sexual fixation is also suggested to be more pronounced in individuals with paedophilia (Bergner-Koether et al., 2024). Around half the current sample reported a sexual interest in minors. Consistent with previous research, escalation in both the severity and frequency of pornography use appeared to be driven by a need for novelty and a pattern of habituation (Wortley et al., 2024), suggesting that a reduction in habituation could support the disruption of offending patterns. Interestingly, participants reported varied pathways to accessing CSEM, aligning with recent findings by Wortley et al. (2024), highlighting the diverse routes into CSEM use and the need for multi-faceted approaches to tackle the availability and accessibility of OCSEA material.

A range of offence-supportive cognitions were reported by participants including justifying the offence, beliefs that children could consent to sex, believing the availability of CSEM reflected its legality, minimising the harm of offending, statements of uncontrollability, and moral disengagement. Cognitive distortions are commonly reported among CSEM users and people who groom minors online (Paquette & Cortoni, 2021; Paquette & Fortin, 2023; Rimer & Holt, 2023). Similarities were seen between the beliefs reported in the current research and implicit theories identified by Bartels and Merdian (2016) including *Self as Uncontrollable, Children as Sex Objects, Unhappy World,* and *Nature of Harm*. Interestingly, some participants experienced cognitive dissonance between their need for sexual gratification and the knowledge that their actions or the material they were accessing were wrong and engaged in a process of moral disengagement to justify their offending. This process of moral disengagement appeared to allow participants to cognitively reframe their behaviour to justify and give permission to their offending. Moral disengagement has yet to be explored in OCSEA literature, however, has been explored in relation to other offending (Niebieszczanski et al., 2015; Shulman et al., 2011). The process of moral disengagement may have implications for prevention strategies. For example, warning messages often seek to provide information advising on the illegality of CSEM based on the presumption that individuals are unaware that it is illegal and harmful (Newman et al., 2024; Prichard et al., 2022). While such messages will be effective for some individuals, it is crucial to recognise that their scope is limited, and different messages need to target those not motivated to prevent harm. Exploring the role of moral disengagement in response to warning messages may prove a useful line of future inquiry.

Treatment was reported to assist participants with developing insight into their offending and to desist from harmful behaviours. While research has not examined reoffending outcomes post-treatment for individuals engaged in OCSEA, within-treatment evaluations have identified improvements pre-post intervention in mental health needs, social skills, cognitive distortions, emotion regulation, and empowering change (Dervley et al., 2017; Gillespie et al., 2018; Middleton et al., 2006). Family and social support post-offending was reported as key for participants, either hindering progress and exacerbating stigma, or providing an avenue of support. Social support is noted as a protective factor in desistance from offending (De Vries Robbé et al., 2015) and is an important area to bolster through intervention.

### Strength and Limitations

This study has several key strengths. By maintaining an inclusive approach to OCSEA behaviour, shared experiences were identified across different forms of online offending compared to exclusively considering CSEM or online grooming like previous studies. We also used flexible participation options to support participant engagement in a safe way across a geographically diverse area, to overcome known recruitment challenges (Ray et al., 2010). The use of a life course narrative approach allowed for the exploration of distal influences, which are often under examined in OCSEA research, and the identification of developmental factors that warrant further investigation. Finally, the use of IPA allowed for an in-depth analysis of individuals’ experiences and pathways into offending. The sample size of nine is relatively large for IPA (Smith et al., 2021) and larger than other IPA studies in the area (Kloess et al., 2019; Sheehan & Sullivan, 2010; Winder & Gough, 2010).

While the study has several methodological strengths, it is also pertinent to acknowledge its limitations. While we maintained a broad eligibility criteria, most participants were involved in online grooming (*n* = 6) and/or CSEM use (*n* = 8). Consequently, not all OCSEA behaviours, such as production of CSEM, live-streaming, or the use of virtual CSEM, are reflected in the sample. The motivations and pathways for these offence categories may therefore differ from current findings. Future research would benefit from studying other types of OCSEA to understand whether they are distinct forms of offending or if they share similar characteristics and treatment needs to those who engage in grooming and CSEM viewing/sharing. Additionally, most (*n* = 8) participants in the study had been adjudicated for their offending, while it is known many individuals who engage in OCSEA remain undetected (Wolak et al., 2014). Research suggests there might be psychological and behavioural differences which explain why some individuals might be detected for offending versus those who avoid detection (Nicol et al., 2024). Future research would benefit from exploring the experiences of undetected individuals to examine if there are any similarities or differences in the factors implicated in the aetiology of their offending. Such research is challenging for various legal and ethical reasons, however, there is an emerging body of research examining self-reported deviant sexual interests and behaviours in community samples which may provide a useful template for continued research (Napier et al., 2024; Seto & Eke, 2015). Additionally, future research should also examine the lived experiences of women who engage in OCSEA. Existing work suggests that women who sexually offend may present with different risk factors (Fortunato et al., 2024), and qualitative research is needed to understand whether similar or distinct distal and proximal factors are implicated in women’s online offending pathways.

### Implications for Practice

The research findings highlight several potential avenues for early intervention for OCSEA offending. First, there is a clear need for consistent and comprehensive sex education in schools to educate young people on healthy intimate relationships and to challenge problematic attitudes and behaviours. Sex education can act as a primary prevention strategy and should include teachings on consent, boundaries, what healthy sexual relationships look like, legal and illegal online behaviours, and the associated harms of online sexual behaviour, including the negative impacts of pornography. Existing sex education programmes are often limited in scope and rarely impact behavioural change (Patterson et al., 2022). Providing effectual sex education to all students represents a primary prevention avenue through providing all individuals with a safe foundational knowledge of healthy behaviours early in life, and the skills needed to manage online sexual behaviour safely. Repeating the process to ensure embedded learning at key developmental milestones has been found to be key in comprehensive sex education programmes, however, they rarely incorporate safe online behaviours (Miedema et al., 2020). Education on the impact and potential harms of pornography should also extend to adults given the identified harms that can occur across the lifespan. Greater research is required on the impact of extreme pornography use, and the harms of continued habituation on attitudes in adults, and whether the impact of such pornography warrants greater restrictions between public and private enterprise.

Possible initiatives at the secondary prevention level are also identified in the current research, through reflections from participants in accessing support services. Secondary prevention for individuals concerned about their behaviour or interests, or a guardian concerned about a minor, may benefit from both educational intervention and referrals for individual intervention and support (Lloyd, 2019), which in turn requires an improvement to the accessibility of self-referral services. Service accessibility could be enhanced through repeated public health promotion campaigns regarding consent, dispelling problematic attitudes, and raising awareness about safe online behaviours in vulnerable groups and/or in online spaces where people might engage in problematic behaviour. Public advertising campaigns have been used in the UK (Newman et al., 2024) and Germany (Beier, 2016) across digital, radio, television, and print media to advertise hotlines and self-referral services and have proven effective in increasing accessibility to the service (Newman et al., 2024), with associated support improving psychological outcomes for attendees (Beier, 2016). The use of prehabilitation programmes as a secondary intervention method have also been trialled, wherein tailored psychological support is offered to individuals who are concerned about themselves or others. Prevention Project Dunkelfeld has been used in Germany (Beier, 2016), and recently the Stand Strong Walk Tall programme has been trialled in New Zealand (Christofferson et al., 2025). However, fear of stigma and social and legal consequences have been found to be significant barriers for men accessing secondary or tertiary support that experience paedophilic interest (Christofferson et al., 2025).

Finally, the current research indicates several areas of potential treatment need for OCSEA offending. Many identified treatment targets are already incorporated into existing interventions, such as mental health support, social competence, improving empathy, and challenging offence supportive attitudes (Dervley et al., 2017; Gillespie et al., 2018). Effects of treatment could potentially be enhanced through inclusion of content on adaptive coping and healthy sexual boundaries, compared to existing programmes which focus primarily on countering avoidance behaviours, or the use of sex as a coping mechanism (Gillespie et al., 2018; Middleton et al., 2006).

### Conclusion

Taken together, the findings from the current research highlight the importance of early modelling experiences, offence supportive attitudes, interpersonal relationships, sexual preoccupation and escalation, and self- and emotion regulation difficulties in the aetiology of OCSEA. The findings also emphasise the potential for primary prevention initiatives to target the provision of education on healthy sexual and interpersonal relationships, and secondary prevention initiatives to provide safe, accessible, and effective psychological support. Further research is needed to explore the pathways into and through a range of OCSEA offending behaviours. Specifically, the relationship between various psychological and environmental factors and engagement in OCSEA across different samples (e.g., correctional, unapprehended) to inform prevention development. Such research would enable a greater understanding of how the offence process unfolds over time and similarities and differences between various offence trajectories.

## Notes

1. Te Reo word meaning separated or alienated.

2. Te Reo word meaning genealogy or ancestry.

3. Te Reo word meaning family.
